# Nano Liposomes Labeled with ^99m^Tc-HMPAO, a Novel Agent for Blood Pool Imaging

**Published:** 2015

**Authors:** Kayvan Sadri, Salimeh Momenypoor, Vahid Reza Dabbagh Kakhki, Ramin Sadeghi, Kamran Aryana, Fariba Johari Daha, Seyed Rasoul Zakavi, Mahmoud Reza Jaafari

**Affiliations:** a *Nuclear Medicine Research Center, Mashhad University of Medical Sciences, Mashhad, Iran.*; b ^*b*^*Biotechcology Research Center, Nanotechnology Research Center, School of Pharmacy, MUMS, Mashhad, Iran. *; c *Nuclear Science and Technology Research Institute, Tehran, Iran.*

**Keywords:** Nano liposomes, ^99m^Tc-HMPAO, Blood Pool Imaging, SPECT, RBC

## Abstract

*In-vitro* labeling of RBC with ^99m^Tc is an intricate procedure and there is always a need for an alternate blood pool imaging agent. The aim of this study was to prepare an effective nano sized liposome (NLs) similar to human RBC for blood pool scintigraphy. This study formulates PEG-NLs and non-PEG-NLs using film method plus high pressure homogenization technique. Biodistribution studies were performed on BALB/C mice 1, 4 and 24 h after tail vein injection of labeled NLs with ^99m^Tc hexamethylpropylene-amine-oxime (^99m^Tc-HMPAO). Planar images were acquired using a 256 × 256 matrix following^99m ^Tc-HMPAO-NLs injection into ear vein of rabbits 1, 2 and 24 h later. SPECT images were obtained 15 minutes after the injection (64 slices, 30 second/projection). The mean diameter, zeta potential and polydispersity index (PDI) of the PEG-NLs and the NLs were (80.88 ± 0.594 nm, -12.5 ± 0.56 mv, 0.158 ± 0.025) and (94.14 ± 0.114 nm, -35.5 ± 0.67 mv and 0.198 ± 0.007), respectively. ^99m^Tc-HMPAO-PEG-NLs showed a significant circulation tracer activity (7.74 ± 1.63%ID/g at 1 h and 4.9 ± 0.77 %ID/g at 4 h), with low liver accumulation (12.07 ± 3.66 %ID/g at 1 h and 14.85 ± 1.3 %ID/g at 4 h). Heart to liver, spleen and background ROIs (region of interests) for ^99m ^Tc-HMPAO-PEG-NLs were 1.25, 4 and 4.28 respectively at 2 h which changed to 1.06, 1.75 and 2.51 respectively at 24 h. The ^99m^Tc-HMPAO-PEG-NLs with a prolonged blood circulation time could be an excellent RBC alternative for scintigraphy and gastrointestinal bleeding.

## Introduction

The most commonly used radiopharmaceutical to evaluate dynamic heart function, vascular malformations and gastrointestinal bleeding is the patient’s own red blood cells (RBC) labeled *in-vitro* or *in-vivo* with ^99m^Tc. The essential needs for a good blood pool imaging agent are; a) long circulation time in blood to present continuous blood pool activity during the time of acquisition, b) minimal accumulation in liver and extravascular space that would interfere with heart blood pool and vascular assessment (1). Labeling of patients’ RBC with ^99m^Tc is not optimal. Apart from risks of blood handling and contamination, drug interference with cell labeling results in poor labeling and suboptimal images (2-3). Although ^99m^Tc is separated from RBC gradually and free ^99m^Tc is taken up by the salivary glands and gastric mucosa and then secreted into gastrointestinal tract which results in poor target to background activity in delayed images (4). This study tries to evaluate nano liposomes as a new agent for blood pool scintigraphy. This technique has the potential to increase patient convenience and decrease labeling pitfalls and blood transferred disease.

Liposomes are spherical phospholipid bilayers that have been investigated for more than 40 years as a drug carrier.

Due to their nontoxic and biodegradable formulation, the clinical interest has increased over liposome application for diagnostic and therapeutic purposes (5-6). In addition advanced progresses in liposome preparation technique lead to the new generation of liposome which has longer circulation time with better biodistribution pattern (7-8).

The major obstacle in clinical application of liposomes is their rapid uptake by phagocytic cells of the mononuclear phagocyte system (MPS). Awasthi, et al. (9), showed that polyethylene glycol (PEG)-liposomes overcome this problem by evading from RES uptake and therefore liver uptake was minimum, spleen uptake was moderate while the amount of circulating liposomes was maximum. Incorporation of hydrophilic PEG in the lipid bilayers induces repulsive interaction with protein plasma, reduced recognition by circulating opsonins and inhibits liposome-induced complement activation (10-11). The development of these long circulating liposomes evolves their clinical usage for scintigraphy visualization of pathological processes such as tumor, infection and blood pool imaging when labeled with a gamma emitter radionuclide (Radioactive liposomes).

There are two basic approaches to this study: the first approach is to design surface modified nano liposomes (NLs) similar to blood cells, therefore reduced host detection rate of liposomes as foreign substances with minimal liver uptake and the lowest liver overlap with heart visualization. The second approach aims to produce biologically inert and completely biocompatible liposomal formulation that they cause practically no toxic or antigenic reactions after IV injection.

The assessment of circulation and tissue distribution of liposomes was performed in mice by labeling liposomes with the lipophilic chelator ^99m^Tc hexamethylpropylene-amine-oxime (^99m^Tc HMPAO) (12). Also scintigraphic imaging was performed in rabbits with planar and SPECT modes using a dual head Gamma camera (e-cam, Siemens, Pennsylvania, USA).

## Experimental

*Materials*

HSPC (hydrogenated soy phosphatidylcholine), DSPE-PEG-2000 (1, 2-distearoyl-*sn*-glycero-3 phosphoethanol amine-*N*- [methoxy (polyethylene glycol)-2000] ), Cholesterol (Chol), 1, 2-distearoyl-sn-glycero-3- [Phospho-rac- (1-glycerol) ] (Sodium Salt) (DSPG), were purchased from Avanti Polar Lipids (Birmingham, USA).Glutathione (GSH), and sephadex G-25 were obtained from Sigma Chemical Co. (St. Louis, MO). HMPAO kit was synthesized and prepared similar to Commercial kit (CERETEC; 0.5 HMPAO and 7.6 µg Sncl_2_). Chloroform and methanol were purchased from Merck (Germany).

^99m^Tc sodium pertechnetate, was obtained from ^99^Mo/^99m^Tc generator (Kimia pakhsh). All chemicals were used without further purification.

*Preparation of Liposome*

PEGylated Nano-liposome (PEG-NLs) and non- PEGylated Nano-liposome (NLs) were prepared by hydration of a thin lipid film followed by high pressure homogenization (13). Briefly, the lipid mixture, HSPC, DSPE-PEG2000 and cholesterol (PEG-NLs, with 12.5: 8: 1.2 molar ratios) and HSPC, cholesterol and DSPG (NLs, 12.5: 8: 2.1 molar ratios) were dissolved in a chloroform-methanol mixture (1:2 volume ratio). A thin-lipid film was formed by removing the solvent on a rotary evaporator under reduced pressure. Liposomes were prepared by rehydrating the lipid film with 100 mM solution of glutathione in phosphate buffered saline (PBS, pH 7) followed by mixing on a vortex mixer for 10 min, sonication at 65ºC for 20 min in a bath type sonicator (Laboratory Supplies Company Inc., Hicksville, NY) under argon. The lipid suspension was subsequently homogenized using EmulsiFlex-C3 high pressure homogenizer (Avestin, Canada) 20,000 PSI for 3 times to obtain homogenous nano-sized lipsomes. Removal of the unencapsulated GSH was performed by overnight dialysis of liposomes at 2–8°C against PBS using a12 kDa dialysis bag.

The particle diameter of each sample was measured in triplicate using Dynamic Light Scattering Instrument (Nano-ZS; Malvern,UK). The zeta potential of liposomes was determined on the same machine using the zeta potential mode as the average of 20 measurements(14).

*Radiolabeling of liposomes*

Liposomes were labeled essentially by the method developed by Phillips, et al.(12). Liposomes (22 µmole/1 ml) were mixed with 370 MBq per 0.5 ml of freshly prepared ^99m^Tc-HMPAO. After 30 min of incubation at room temperature, liposomes were separated from any free ^99m^Tc by passing through Sephadex G-25. The column was eluted with Dextrose 5% and the labeling efficiency was determined by counting liposomes before and after purification with a dose calibrator (Capintec, CRC-15R).

*Radiolabeling Stability in Human Serum*

The radiolabeling stability of the PEG-NLs and NLs was evaluated in the presence of human serum at 37°C for 24 hours. After different incubation periods (1, 2 and 24 h), labeling stability of liposomes was measured using ITLC-SG/MEK (silica gel as stationary phase and Methyl Ethyl Ketone and as mobile phase)

*Animal biodistribution study*

The animal experiments were performed according to the NIH Animal Use and Care Guidelines and were approved by the Institutional Animal Care Committee of Mashhad University of Medical Sciences. For biodistribution studies, BALB/C mice (3 groups each with 3 mice) were injected through a lateral tail vein, with ^99m^Tc-HMPAO-nano liposomes (7.4 MBq, 200 µCi). The BALB/C mice were sacrificed for each time studied; 1, 4 and 24 h after injection. The blood was collected and organs of interest (muscle, intestine, heart, lungs, blood, spleen, kidneys, liver, stomach, bone, tail and thyroid) were dissected, weighed and counted for ^99m^Tc-HMPAO-nano-Liposome activity in a well gamma counter (Delshid, DL100). The percent injected dose per gram was calculated by dividing the accumulated activity in the organ to its weight (%ID/g, Injected Dose per gram organ).

*Animal Scintigraphic imaging*

White rabbits (1.5-2 kg) were physically restrained in a fixation tool and injected via the ear vein with 0.5 ml of ^99m^Tc-HMPAO nano-Liposome (34-74 kBq, 1-2 mCi). Planar and SPECT imaging were obtained using a Siemens Dual head Gamma Camera equipped with a low energy high-resolution collimator and peaked at 140 keV with ± 20% window. Images were acquired using a 256 × 256 matrix for a total of 300,000 counts immediately after injection and at 1, 2 and 24 h later with a zoom factor of 2. For all time-points the animals were physically restrained while positioned over the detector. 

Region of interest (ROI) was drawn around the heart, liver, spleen and background area and total count, mean count per pixel as well as maximum count per pixel was recorded. Geometric mean count and geometric maximum count were calculated from anterior and posterior views for each organ.

*Statistical analysis*

Data are expressed as mean ± standard deviation. Means were compared using Student’s t-test. The *p *values of less than 0.05 were considered statistically significant.

## Results and Discussion

*Liposome characterization*

The mean diameter, zeta potential and polydispersity index (PDI) of the PEG-NLs were 80.88 ± 0.594 nm (n = 3), -12.5 ± 0.56 mv and 0.158 ± 0.025, respectively. The mean diameter, zeta potential and PDI of the NLs were 94.14 ± 0.114 nm (n = 3), -35.5 ± 0.67 mv and 0.198 ± 0.007, respectively. Both liposomal formulations had almost the same size (Table 1). The zeta potential of NLs was significantly more negatively charged compared to PEG-NLs due to the presence of negatively charged phospholipid (DSPG) in the formulation of NLs. Both formulations had a monomodal distribution. A low PDI value with a monomodal peak represents a narrow particle size distribution for these liposomes.

**Table 1:** Formulation and characterization of PEG-NLs and NLs using film method plus high pressure homogenization technique

	**PEG-NLs** **HSPC, DSPE-PEG2000 and cholesterol with molar ratio of 12.5: 8: 1.2**	**NLs** **HSPC, cholesterol and DSPG with molar ratio of 12.5: 8: 2.1**
Z-Average(nm)	80.88 ± 0.594	94.14 ± 0.114
PDI	0.158 ± 0.025	0.198 ± 0.007
Zeta Potential(mv)	-12.5 ± 0.56	-35.5 ± 0.67

*Radiochemical Purity and Stability*

**Figure 1:**
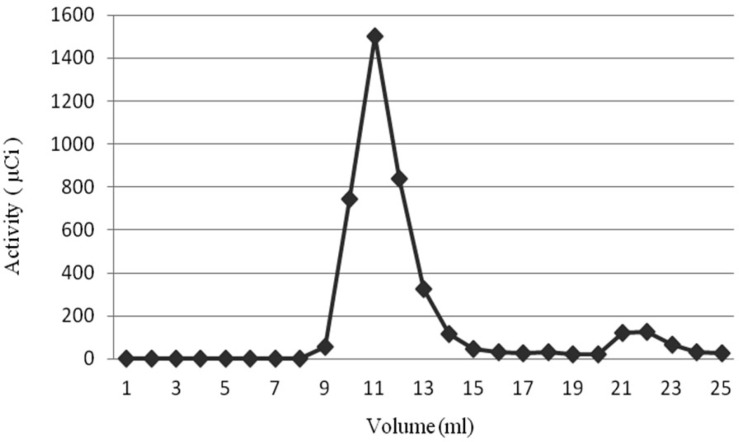
Size exclusion chromatography of PEG-NLs labeled with ^99m^Tc-HMPAO on Sephadex G-25. Elution volumes of tube 10-13 belong to ^99m^Tc-HMPAO-PEG-NLs and elution volumes of tube 20-23 belong to lipophilic ^99m^Tc-HMPAO

Radiochemical Purity of PEG-NLs and NLs, after liposomes incubation for 30 minutes with 370 MBq ^99m^Tc-HMPAO, was 91.55 ± 1.57% and 93.8 ± 2.43%, respectively. This high Radiochemical purity was obtained by passing the liposomes through Sephadex G25 which separated nano liposomes entrapping ^99m^Tc-HMPAO from free ^99m^Tc-HMPAO (Figure 1). In order to make sure of the ^99m^Tc-HMPAO stability in the liposome, *in-vitro* stability test was performed by exposing the labeled liposome to human serum plasma. As shown in Table 2, the labeling stability of PEG-NLs obtained by ITLC up to 4 hours was more than 95% and 78% at 24 hours. The labeling stability of NLs was more than 65% at 4 hours and 50% at 24 hours.

**Table 2:** Liposome stability studies in human serum media to 24 h (ITLC-SG as stationary phase and Methyl Ethyl Ketone as mobile phase). ^99m^Tc-NLs remains at the origin and free Pertechnetate migrate to the solvent front. The labeling stability was calculated by dividing ^99m^Tc-NLs activity to ^99m^Tc-NLs + free Pertechnetate

**Incubation times (hours)**	**1**	**2**	**4**	**24**
Labeling stability of ^99m^Tc-PEG-NLs	96.90 ± 0.64	94.34 ± 0.56	95.38 ± 0.43	78.54 ± 0.34
Labeling stability of ^99m^Tc-NLs	75.80 ± 0.78	71.65 ± 1.3	65.72 ± 0.78	50.96 ± 1.52

*Tissue Distribution in Mice*

The biodistribution of the radiolabeled liposomes were performed in mice at different time points (n = 3 for each time). As shown in Table 3, ^99m^Tc-HMPAO-PEG-NLs had a low liver uptake (12.07 ± 3.66 %ID/g, 14.85 ± 1.3, 6.59 %ID/g ± 1.29%ID/g at 1, 4 and 24 h, respectively) and a high blood pool uptake (7.74 ± 1.63 %ID/g, 4.9 ± 0.77 %ID/g, 0.92 ± 0.43%ID/g at 1, 4 and 24 h, respectively). 99mTc-HMPAO-NLs had much higher liver accumulation (36.81 ± 6.56 %ID/g, 26.83 ± 2.49 %ID/g, 20.7 ± 2.79 %ID/g at 1, 4 and 24 h; respectively). The spleen uptake was also elevated for these NLs (28.63 ± 5.62 %ID/g, 48.63 ± 5.36 %ID/g, 63.55 ± 5.89%ID/g at 1, 4 and 24 h; respectively). This high accumulation may be the result of nano-sized PEGylated liposomes (less than 100 nm) which cleared more rapidly from the spleen (15).

**Table 3:** Tissue distribution of ^99m^Tc-HMPAO-PEG-NLs and NLs in mice (%ID/g, n = 3) at 1, 4 and 24 hours after injection of ^99m^Tc-HMPAO-NLs (7.4 MBq, 200 µCi) (*p* < 0.05

**Tissues**		^99m^ **Tc-PEGNLs (%ID/g)**			^99m^ **Tc-NLs (%ID/g)**	
	1 h	4 h	24 h	1 h	4 h	24 h
Muscle	0.39±0.9	0.07±0.02	0.29±0.09	0.36±0.25	0.14±0.2	0.42±0.12
Intestine	0.24±0.11	0.2±0.17	0.46±0.05	0.38±0.11	0.17±0.04	0.28±0.04
Heart	0.62±0.3	0.31±0.11	0.32±0.39	0.91±0.32	0.27±0.13	0.44±0.6
Lungs	0.66±0.5	0.48±0.13	0.89±0.03	1.03±0.47	0.55±0.1	0.39±0.02
Blood	7.74±1.63*	4.9±0.77*	0.92±0.43*	15.06±2.12*	7.57±1.08*	0.38±0.01*
Spleen	37.99±2.92*	29.85±1.01*	14.89±4.03*	28.63±5.6*	48.63±5.36*	63.55±5.89*
Kidneys	0.83±0.35	0.64±0.14	1.08±0.25	1.49±1.03	1.02±0.05	0.79±0.11
Liver	12.07±3.66	14.85±1.3	6.59±1.29*	36.81±6.56	26.83±2.49	20.7±2.79
Stomah	0.23±0.5	0.19±0.04	0.4±0.24	0.22±0.3	0.13±0.09	0.35±0.13
bone	0.25±0.2	0.07±0.04	0.56±0.38	0.2±0.17	0.11±0.03	0.23±0.01
Tail	0.1±0.06	0.43±0.06	3.19±1.72	0.29±0.1	4.14±2.6	4.7±3.27
thyroid	0.92±0.5	0.24±0.2	0.96±0.64	1.13±1.07	0.53±0.16	0.89±0.76

* Significant (*p* value < 0.05)

*Scintigraphic Studies in Rabbit*

**Figure 2:**
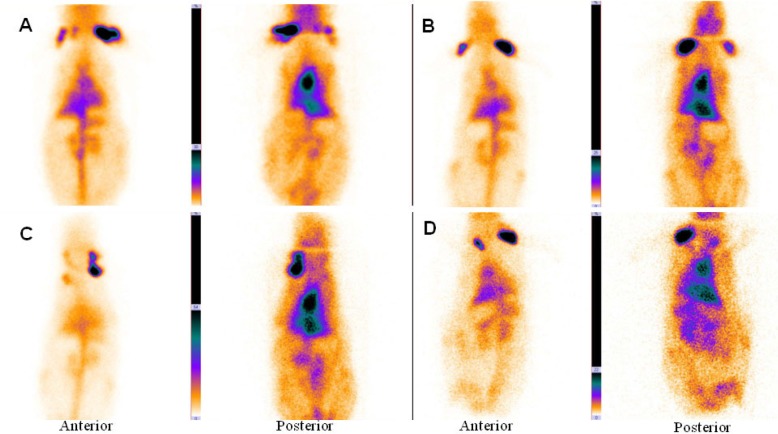
Anterior and posterior images of rabbit immediately (A), 1 h (B), 2 h (C) and 24 h (D) after injection of ^99m^Tc-HMPAO-PEG-NLs (34-74 kBq, 1-2 mCi in 0.5 ml). Planar images were acquired using a 256 × 256 matrix for a total of 300,000 counts with a zoom factor of 2. The main vessels (Aorta), heart and liver was clearly seen in the anterior and posterior images of rabbit for 24 h.

The main vessels (Aorta), heart and liver was clearly seen in the anterior and posterior images of rabbit. Figure 2A, B shows planar imaging of rabbit in the early times (immediately and 1 h) after ^99m^Tc-HMPAO-PEG-NLs injection.

**Figure 3:**
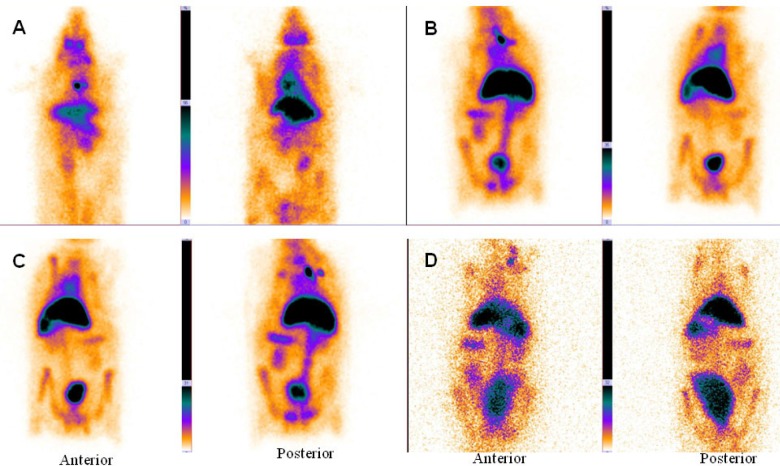
Anterior and posterior images of rabbit immediately (A), 1 h (B), 2 h (C) and 24 h (D) after injection of ^99m^Tc-HMPAO-NLs (34-74 kBq, 1-2 mCi in 0.5 ml). Planar images were acquired using a 256 × 256 matrix for a total of 300,000 counts with a zoom factor of 2. ^99m^Tc-HMPAO-NLs showed a high liver uptake and low heart accumulation

In the delayed images obtained 2 and 24 hours later (Figure 2C, D), heart and liver had high radiotracer activity. This is a proof to prolong circulation profile of ^99m^Tc-HMPAO-PEG-NLs in the blood stream. In the delayed images, there was no uptake in the kidneys and bladder wall which is important in the diagnosis of lower gastrointestinal bleeding. Scintigraphy images obtained with ^99m^Tc-HMPAO-NLs (Figure 3) showed a high liver uptake and low heart accumulation. Although these liposomes had a high blood pool circulation time, but their *in-vivo* stability was too low to be useful for *in-vivo* studies which is in agreement with *in-vitro* stability test (Table 2).

Total count and geometric mean count per pixel was calculated at 0, 2 and 24 h post injection for ^99m^Tc-HMPAO-PEG-NLs. Heart to liver, heart to spleen and heart to background ROIs were 1.25, 4 and 4.28 immediately, 1.12, 4.33 and 3.25 at 2 hours, and 1.06, 1.75 and 2.51 at 24 hours post injection, respectively.

The main aim of this study was to prepare an effective nano sized liposome similar to human red blood cell (RBC) for blood pool scintigraphy. By optimizing the liposomes characteristics such as vesicle size, lipid formulation and hydrophilicity of liposome surface (7), we prepared an effective formulation of nano sized PEG-NLs and non-PEG NLs which were homogeneous in size (PDI less than 0.2) and similar to RBC in having prolonged circulation time for diagnostic purpose. Both formulations were negatively charged that prevents their aggregation during the storage.

Since liposomes used in this study are composed of natural biomolecules (HSPC, cholesterol, DSPE-mPEG 2000 and DSPG), they are biodegradable and essentially non-toxic. HSPC, cholesterol, DSPE-mPEG 2000 and DSPG are applied in the formulation of the available commercial liposomal formulations (e.g. AmBisome^TM^ and Doxil^TM^) with a very good safety profile (16-17).

**Figure 4:**
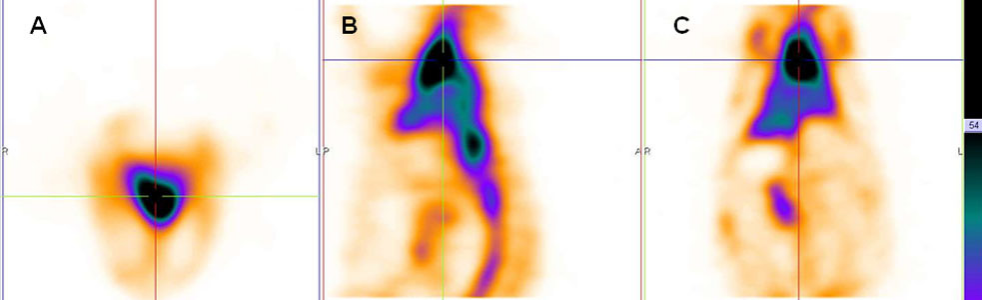
SPECT images of rabbit (A. transverse B. sagittal C. coronal) were obtained 15 minutes after injection of ^99m^Tc-HMPAO-PEG-NLs (64 slices, 30 second/projection).

In this study, PEG-NLs incorporated with 5.5 mol% DSPE-mPEG 2000 have a mean diameter of 80.88 ± 0.594 nm. In mice tissue distribution study, blood pool circulation was very high 7.74 ± 1.63 %ID/g at 1 h and 4.9 ± 0.77 %ID/g at 4 h (*p *< 0.05) (Table 3). Compared to human Red blood Cell labeled with ^99m^Tc (4), there was no significant activity in thyroid or stomach showing the *in-vivo* stability of ^99m^Tc-HMPAO-PEG-NLs. The high labeling efficiency guarantee a high target to background ratio and optimal image quality which have an effect on scan interpretation (Figure 2, 4). This result was in harmony with the *in-vitro* stability test in human serum albumin (Table 2). ^99m^Tc-HMPAO-NLs prepared in this study had a high blood pool circulation in mice 15.06±2.12 %ID/g at 1 h and 7.57±1.08 %ID/g at 4 h (*p *< 0.05) (Table 3). Although NLs had a high blood pool activity, but their elevated accumulation in liver and spleen underestimate their clinical usage as a blood pool imaging agent (Figure 3).

**Figure 5:**
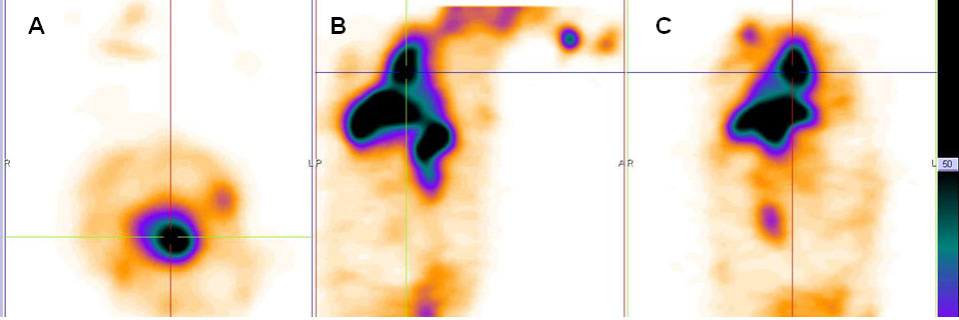
SPECT images of rabbit (A. transverse B. sagittal C. coronal) were obtained 15 minutes after injection of ^99m^Tc-HMPAO-NLs (64 slices, 30 second/projection).

Scintigraphy images obtained in rabbits approved the long blood pool circulation time of ^99m^Tc-HMPAO-PEG-NLs (17). Heart-to-liver and heart-to-spleen and heart-to-background ratios were determined from region of interest (ROI) analysis of zoomed static images. The ROI values for heart to liver were steady (1.25, 1.12 and 1.06 at 0, 2 and 24 h post injection, respectively) which shows high PEG-NLs uptake in the heart to 24 h. For cardiac functional studies, the relationship of the distribution of the agent in the heart compared to the liver activity is the most important factor. In the early planar images (Figure 2A) and SPECT images obtained 15 minutes after labeled PEG-NLs administration to the rabbit (Figure 4), high heart activity is an evident for the increased residence of labeled liposome in the blood pool. Low liver uptake during this time also helps in better heart visulaization. Although NLs had a long circulation time in blood, but their high accumulation in the liver overlap the heart visualization(2) (Figure 3, 5).

In the delayed images obtained with ^99m^Tc-HMPAO PEG-NLs (Figure 2D , 24 h later), there was also some uptake in the abdominal region that demonstrates the labeled liposome metabolism by liver. In all the early and delayed images (Figure 2), there was no evidence of kidney uptake. Goins, *et al*.(2) studies also demonstrate lower kidney activity following PEGylated liposome injection compared to both in-vitro and in-vivo ^99m^Tc red blood cells labeling. This mild kidney uptake in delayed scintigraphy is so essential in detection of lower gastrointestinal bleeding region which needs delayed imaging. In addition, PEG-NLs labeled with ^99m^Tc-HMPAO are unlikely to be affected by administered medications to the patient that can interfere with the labeling and stability of ^99m^Tc-RBC (18).

In early and delayed images obtained with ^99m^Tc-HMPAO-NLs (Figure 3), there was an increased bladder activity over time. This is due to the instable NLs and the leakage of ^99m^Tc-HMAPO from the liposomes and its excretion through kidney and bladder (7). These results show us the priority of PEG-NL over non-PEG NLs for GI bleeding scintigraphy due to their excellent *in-vivo* stability (Table 2). The lack of dissociation of the ^99m^Tc from PEG-NLs results in better understanding of cardiac functionality and counting statistics for cardiac studies.

## Conclusion

This study demonstrated that PEG nano sized liposomes (PEG-NLs) could be a valuable radiopharmaceutical for blood pool imaging with no need for blood withdrawal and potential complications. It has an easy labeling procedure and provides the physician with better understanding of blood pool scintigraphy.
